# Heterogeneity of B Cell Functions in Stroke-Related Risk, Prevention, Injury, and Repair

**DOI:** 10.1007/s13311-016-0460-4

**Published:** 2016-08-04

**Authors:** Uma Maheswari Selvaraj, Katherine Poinsatte, Vanessa Torres, Sterling B. Ortega, Ann M. Stowe

**Affiliations:** Department of Neurology and Neurotherapeutics, UT Southwestern Medical Center, 6000 Harry Hines Blvd, MC8813, Dallas, TX 75390 USA

**Keywords:** B lymphocyte, Breg, Hypertension, Atherosclerosis, Ischemic tolerance, Autoreactivity

## Abstract

It is well established that post-stroke inflammation contributes to neurovascular injury, blood–brain barrier disruption, and poor functional recovery in both animal and clinical studies. However, recent studies also suggest that several leukocyte subsets, activated during the post-stroke immune response, can exhibit both pro-injury and pro-recovery phenotypes. In accordance with these findings, B lymphocytes, or B cells, play a heterogeneous role in the adaptive immune response to stroke. This review highlights what is currently understood about the various roles of B cells, with an emphasis on stroke risk factors, as well as post-stroke injury and repair. This includes an overview of B cell functions, such as antibody production, cytokine secretion, and contribution to the immune response as antigen presenting cells. Next, evidence for B cell-mediated mechanisms in stroke-related risk factors, including hypertension, diabetes, and atherosclerosis, is outlined, followed by studies that focus on B cells during endogenous protection from stroke. Subsequently, animal studies that investigate the role of B cells in post-stroke injury and repair are summarized, and the final section describes current B cell-related clinical trials for stroke, as well as other central nervous system diseases. This review reveals the complex role of B cells in stroke, with a focus on areas for potential clinical intervention for a disease that affects millions of people globally each year.

**Electronic supplementary material:**

The online version of this article (doi:10.1007/s13311-016-0460-4) contains supplementary material, which is available to authorized users.

## Introduction

Nearly 800,000 people in the USA experience a new or recurrent stroke every year, making stroke the fifth leading cause of death and a major cause of long-term adult disability in the US [1]. Tissue plasminogen activator is currently the only Food and Drug Administration (FDA)-approved post-stroke therapeutic [2], but delivery is required within the first 3 to 6 hours of stroke onset. Therefore, identifying mechanisms that contribute to the evolution of stroke-related injury and repair—occurring over the course of days and weeks—could expand the available window for therapeutic interventions. As highlighted by this special issue, recent focus on understanding the role of the immune system following stroke has identified several mechanisms, involving both the adaptive (e.g., B and T cells) and innate (e.g., monocytes, microglia) immune systems, that could develop into efficacious therapeutic targets. This review focuses on the roles of B cells in stroke risk and post-stroke injury and repair, with an emphasis on potential clinical interventions.

## A Review of B Cell Functions

### B Cells as Antibody Producers

**Fig. 1:**
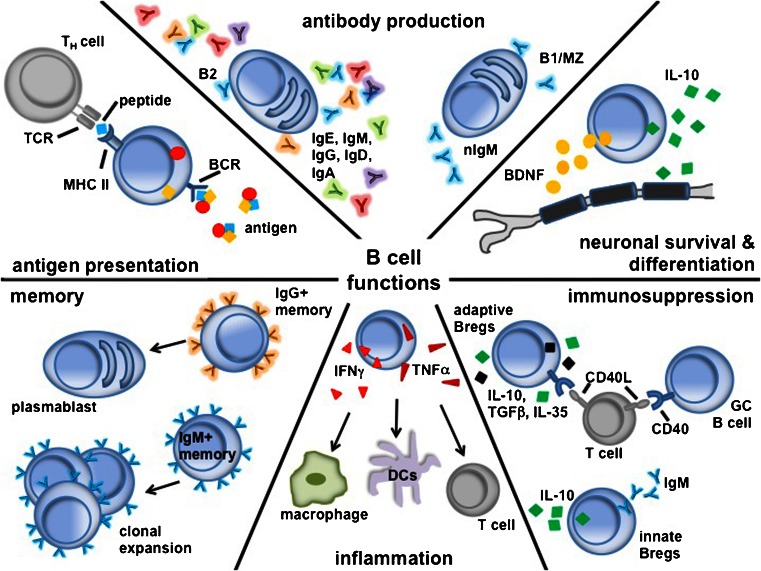
Summary of various B cell functions. T_H_ = T helper; TCR = T cell receptor; MHC = major histocompatibility complex; BCR = B cell receptor; nIgM = natural IgM; MZ = marginal zone; IL = interleukin; BDNF = brain-derived neurotrophic factor; IFN = interferon; TNF = tumor necrosis factor; DC = dendritic cell; Breg = regulatory B cell; TGF = transforming growth factor; GC = Germinal center

Figure 1 pictorializes the different aspects of B cell function summarized in this section. B lymphocytes (i.e., B cells) play a significant role in humoral immune responses. Naïve B cells express the primary effector molecules IgM and IgD. Upon activation, B cells switch their expression to a high-affinity, antigen-specific IgG antibody and differentiate into either memory cells or primary plasma cells (see review [3]). The “switching” to high-affinity antibodies is dependent on interactions with CD4 T cells (i.e., helper T cells), expression of CD40 on B cells, and contact with surrounding cytokines such as interferon IFN-γ or interleukin IL-4. These linked recognitions further activate both T and B cells to proliferate and clonally expand.

The spleen is compartmentalized into different areas, such as follicular regions (mainly contains B cells), the marginal zone (MZ) surrounding the follicles (consists of T cells, macrophages, and B cells), the red pulp (functions as a red blood cell disposal site), and the paracortical region (consists mostly of T cells). Upon B cell activation in the spleen, there is an extrafollicular response in splenic regions, such as the red pulp and a follicular germinal center response [4, 5]. In the extrafollicular response, B cells proliferate and differentiate into short-lived, antibody-secreting plasmablasts [6]. Some of the activated B cells enter the B cell follicles and divide rapidly to form germinal centers. B cells entering germinal center reactions are known as B2 cells [7]. The germinal center provides the optimal microenvironment for various B cell processes, including proliferation, somatic hypermutation, affinity maturation, and isotype switching, all of which are essential for generating antibody diversity. B cells rearrange their DNA and alter the immunoglobulin heavy chain constant region (i.e., class-switch recombination or isotype switching) to induce different effector functions (IgA, IgG, IgM, IgD, IgE). The germinal center consists of 2 areas, a dark zone comprised of rapidly proliferating B cells, and a light zone where B cells interact with follicular dendritic cells and CD4 T cells. B cells in the light zone compete for survival signals from these dendritic cells and T cells, and are susceptible to apoptosis unless selected and expanded. This process of selective survival of B cells is termed “affinity maturation” [8]. B cells then either differentiate into plasma cells, which migrate to the bone marrow, where they continue producing higher-affinity and isotype-switched antibodies, or they differentiate into memory B cells.

Other heterogeneous populations of B cells, like B1 cells and MZ B cells, also produce antibodies to play an essential role in innate sensing of pathogens. B1 cells are generated during fetal and neonatal developmental stages and are mainly present in peritoneal and pleural cavities [9]. These innate-like B1 cells secrete broad reactivity and low-affinity natural IgM (nIgM) antibodies. In addition, MZ B cells, which are mainly present in the MZ of the spleen, also secrete nIgM [10]. These antibodies typically bind pathogens or receptors to act as their ligand (see review [11]), or activate the complement system, leading to the release of anaphylatoxins, C5a and C3a. Release of anaphylatoxins increases secretion of proinflammatory cytokines and attracts other effector cells to the site of inflammation. Polyreactive, nIgM antibodies bind and thereby coat apoptotic cells to also increase the clearance of dying or dead cells [12, 13].

### B Cells as Antigen-Presenting Cells

B cells in the secondary lymphoid organs, like spleen and lymph nodes, are exposed to antigens that either enter through the afferent lymphatics, or are presented on the neighboring cell surface [14]. The B cell receptor (BCR) is an immunoglobulin on the B cell plasma membrane, which binds antigens with varying affinity, resulting in an engulfment of the antigen. The BCR works in concert with downstream signaling cascades, leading to the transcription of genes necessary for B cell function (see review [15]). The antigen, along with the BCR, is internalized in endosomal and lysosomal vesicles and the antigen is processed into peptide fragments. Accessory enzymes like proteases aid the assembly of these peptide fragments onto major histocompatibility class (MHC)-II molecules, which then traffic to the B cell’s surface to become an antigen-presenting cell (APC). The peptide fragments bound to MHC II molecules are presented to CD4 T cells for further antigen-specific T cell receptor interactions [16].

### B Cells as Cytokine Producers

Apart from antibody production and antigen presentation, B cells also play a major role in modulating immune responses through cytokine secretion, as demonstrated through various animal models, especially those for autoimmune [17–19] and inflammatory [12] diseases. B regulatory cells (Bregs) suppress the immune system through the production of anti-inflammatory cytokines such as IL-10, IL-35, and transforming growth factor (TGF)-β. Bregs indirectly exert suppressive activity in a cell contact-dependent manner through the expression of inhibitory molecules, including CD40, on their surface. Bregs inhibit the expansion of inflammatory and autoreactive T cell and B cells [20–22], but require activation for cytokine production [23, 24]. Innate Bregs, derived from B1 cells, rapidly produce high amounts of IL-10 and IgM antibodies [25], while adaptive Bregs display antigen specificity and are activated by both BCR and CD40 stimulation [21]. In addition to innate and adaptive Bregs, MZ precursor B cells secrete IL-10 exclusively in response to CD40 stimulation [26].

Apart from the immunosuppressive cytokines like IL-10 and TGF-β, B cells also secrete proinflammatory cytokines, including tumor necrosis factor (TNF)-α and IFN-γ. Expression depends on the inflammatory environment or the level of anti-inflammatory cytokines such as IL-4, IL-5, and IL-13 [27]. The overall function of these cytokines is to direct Ig isotype production (i.e. IgG1 versus IgG4) [28]. Additionally, activated B cells from both humans and mice have been shown to produce neurotrophic growth factors, such as brain-derived neurotrophic factor (BDNF) and nerve growth factor (NGF), to promote the survival and differentiation of neuronal populations during central nervous system (CNS) injury [29–31].

### Memory B Cells

After the initial immune response to invading pathogens, the germinal center B cells, which have undergone several rounds of positive selection (see “Tolerance Mechanisms in B Cells”), differentiate either into antibody-secreting plasma cells or memory B cells. Long-lived plasma cells home to the bone marrow, where they secrete antibodies and act as a first line of defense upon reinfection [32–34]. Memory B cells are also antigen specific, but while they express surface immunoglobulin, they do not secrete antibody at a high rate (see review [35]). Memory B cells are derived from the B cells that have undergone affinity maturation and isotype selection. Memory B cells are heterogeneous in nature, with subtypes including traditional germinal center-derived B cells, germinal center-independent memory B cells, and IgM class-switched memory B cells [36–38]. IgG^+^ memory B cells differentiate into plasmablasts, while IgM^+^ memory B cells expand during germinal center reactions [39, 40]. Chronic infection exhausts memory B cells and limits functionality [41].

### Tolerance Mechanisms in B Cells

Early B cell development occurs within the bone marrow where B cells undergo both positive and negative selection, as bone marrow stromal cells provide signals to lymphoid progenitor cells through cytokine secretion and cell–cell contacts (see review [42]). These interactions prime progenitors to differentiate and commit to various lineages and start the expression of recombinase enzymes during both early and late B cell developmental stages [43]. Recombinase enzymes are involved in somatic recombination processes, which lead to either productive or non-productive B cells based on the presence of a functional heavy and light chain. B cells with unsuccessful rearrangements and improper immunoglobulin surface expression undergo apoptosis [44]. However, when B cells successfully express the cell surface receptor, signaling through the antigen receptor provides survival signals. Furthermore, multiple checkpoints during B cell development eliminate strongly self-reactive cells.

At the first checkpoint, B cell precursors expressing immunoglobulin heavy chain (IgH) are subjected to the surrogate light chain’s maintenance [45]. The surrogate chain checks how well IgH can pair with the immunoglobulin light chain (IgL) and, if paired appropriately, form the pre-B cell receptor. The light chain rearrangements, which are more complex, occur in 4 stages involving the paternal and maternal k and l loci [45, 46]. Together, B cells with a BCR comprised of light chain and heavy chain are presented with autoantigens and reach another checkpoint. Higher-affinity autoreactive B cells are deleted, while lower-affinity autoreactive B cells, particularly gut- and lung-associated B1 cells, enter the peripheral autoreactive cell pool [45]. B cells that do not recognize autoantigens home to the B cell follicles of spleen and lymph nodes to become mature B2 cells [45].

Thus, through receptor editing, B cells undergo several rounds of rearrangements, followed by positive and negative selection to ensure minimal loss of B cells while eliminating any potentially pathogenic, high-affinity, autoreactive cells. Nearly 75% of early immature B cells in humans are autoreactive, so receptor editing removes a third of these cells from the autoreactive repertoire [47]. This optimizes the chance of survival for the majority of pre-B cells, allowing them to mature into naïve B cells. However, if the initial autoreactive chains are not properly deleted during receptor editing, these B cells can revert and express the original autoreactive receptor and induce autoimmune responses [48]. With respect to autoimmune diseases, B cells are usually considered to be pathogenic owing to the secretion of autoantibodies that exacerbate tissue damage [49, 50]. Apart from secreting autoantibodies, B cells may also contribute to autoimmune disease through pro-inflammatory or anti-inflammatory cytokine production, presentation of self-antigens, regulation of other immune cells, and development of ectopic germinal centers (see reviews [51, 52]).

### Downstream BCR Signaling

BCR signaling determines the selective survival of B cells based on their antigen specificity. The BCR is complexed with Igα and Igβ heterodimers [53]. When the BCR is cross-linked upon binding a receptor-specific antigen, tyrosine residues (e.g., Syk, Lyn) in the cytoplasmic BCR complex are phosphorylated by kinases to assemble adaptor proteins, kinases, guanine exchange factors, and G proteins (see reviews [54, 55]). Signal transduction processes that occur after antigen recognition by the BCR determine the magnitude of the BCR signaling. The strength of these signal transduction processes is, in turn, determined by the surface receptor density, valence, and antigen affinity of the BCR. Syk phosphorylates the Igα and Igβ complexed with the BCR, and Lyn forms a complex with the costimulatory B7 molecules and surface CD19 to reduce the activation threshold [52, 56–58]. Multiple pathways result in BCR signal transduction, including second messenger signaling and mitogen activated protein kinase pathway activation. These second messengers further activate downstream transcription factors essential for B cell function and calcium fluxes [59]. Ultimately, the B cell state and maturation stage, together with the type of antigen activating the BCR, decides the specific pathway activated downstream.

Apart from the positive signaling on antigen recognition, inhibitory receptors such as Fcγ receptors, programmed cell death 1 (CD28 homolog), and CD31 limit the intensity and duration of B cell activation through negative regulatory functions [60]. These are essential for restricting responses to foreign pathogens and discriminating against autoantigens. The disruption of these inhibitory receptors and other B cell intrinsic signaling components have been implicated in the development of autoimmune responses [61]. As discussed previously, apart from antigen recognition and binding, B cells further require secondary signals from CD4 T cells to mount a successful antigen response. MHC-II on the B cell surface binds its respective CD4 T helper cell to induce the secretion of cytokines. These cytokines trigger B cell proliferation and other downstream differentiation processes. Therefore, autoreactive B cells that escape all tolerance checkpoints and manage to enter the periphery can reach a state of unresponsiveness called anergy. Anergic B cells cannot sustain the initial antigen recognition owing to the lack of co-stimulation from CD4 T cells [47].

## B Cells Contribute to the Development of Stroke Risk Factors

**Fig. 2:**
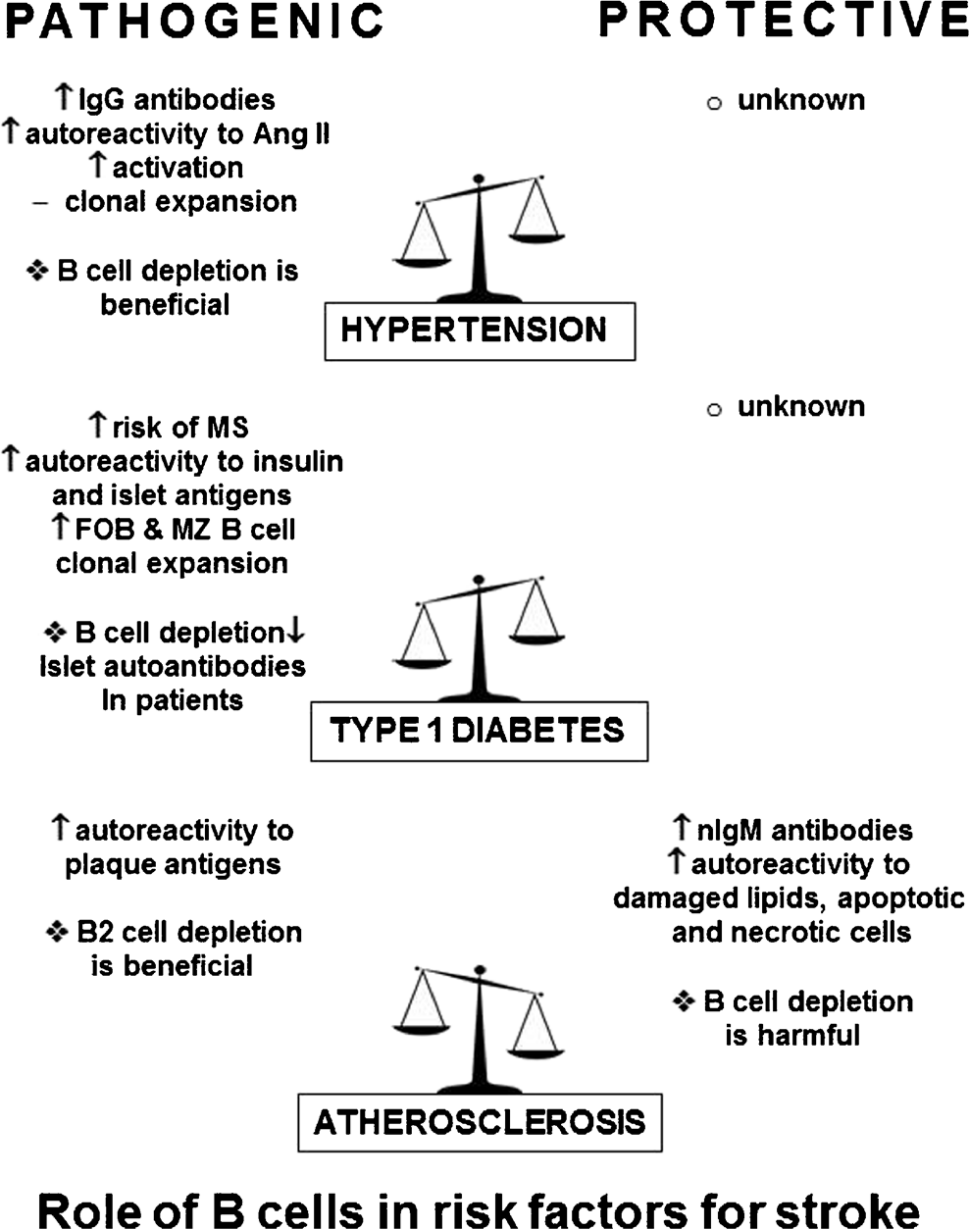
Role of B cells in risk factors for stroke Ang II = angiotensin II; MS = multiple sclerosis; FOB = Follicular B cells; MZ = marginal zone; nIgM = natural IgM

Clinical studies show that particular risk factors, including disease, lifestyle, sex, and age, increase the likelihood of stroke [62]. Of these risk factors—hypertension, diabetes, and atherosclerosis—are known to be potent predictors of stroke incidence, outcome, and recurrence [62]. These diseases have all been linked to changes in B cell function and phenotype, with much of the research suggesting that B cells contribute to disease development and progression [63–66] (as summarized in Fig. 2). Despite the largely deleterious effect of B cells, newer studies identify particular B cell subsets that may also be protective [65–68]. In addition to these diseases, other factors (e.g., sex, obesity, age) contribute to stroke prevalence [62], and induce both beneficial and detrimental changes to B cells [69–71], but are not specifically reviewed in this section.

Many stroke risk factors are highly comorbid [72]. For example, hypertension and diabetes are the leading risk factors for atherosclerosis, and worsen associated disease outcome [72, 73]. In line with this finding, one study showed that 57% of patients with stroke had multiple risk factors, including hypertension and diabetes, and these risk factors were also associated with increased incidence of secondary stroke [74]. The high frequency of comorbid stroke risk factors has led to the development of the “common soil” hypothesis, which suggests shared disease mechanisms for hypertension, diabetes, and atherosclerosis [75, 76], including disease-induced immune dysregulation [75]. This section will review the current research regarding B cells in hypertension, diabetes, and atherosclerosis, including B cell production of autoantibodies to disease-associated antigens as a common mechanism to these major stroke risk factors [64, 65, 77].

### High Blood Pressure and Hypertension

Approximately 80 million adults in the US have hypertension (systolic blood pressure ≥ 140 mmHg) with the prevalence of this disease, and associated deaths, on the rise [62]. At the time of first stroke, approximately 77% of individuals have high blood pressure, and prehypertension is a robust predictor of future strokes [62]. For nearly 50 years, it has been known that the immune system contributes to the progression of this disease. During hypertension, there is dysregulation of the adaptive immune system, leading to global inflammation and oxidative stress in the kidneys and vasculature [78]. This inflammation has largely been attributed to a greater activation of proinflammatory T cells, suppression of regulatory T cells, and increased cytokine production [78].

Despite evidence that the immune system plays an important role in hypertension, the contributions of B cells have only recently come to light [63]. B cells isolated from patients with hypertension exhibit enhanced proliferation and activation in response to *in vitro* stimulation compared with normotensive individuals [79, 80]. Murine studies confirmed that immunodeficient mice that lack B cells and T cells have attenuated disease in response to angiotensin-II (Ang-II), a common rodent model of hypertension [81, 82]. B cells are also critical for the development of hypertension, as pharmacologic depletion of B cells protects against Ang-II-induced increases in systolic blood pressure, while adoptive transfer of naïve B cells restores the development of disease [63]. Additionally, B cell-deficient mice had fewer macrophages and decreased stiffening in the aorta, which is clinically an independent predictor of fatal stroke [83].

Hypertension-induced antibody production may also play a key role in pathogenesis. In hypertensive mice, there are approximately twice as many plasma cells and plasmablasts, as well as greater levels of circulating IgG and IgG deposits in the aorta, compared with wild-type (WT) mice [63]. Multiple studies corroborated that patients with hypertension have increased serum levels of IgG [84, 85], and immortalized B cells from patients have greater IgG production *in vitro* [79]. Patients with hypertension also present with IgG autoantibodies targeting Ang-II receptors [77, 86], with antibody titers correlated to disease severity [87]. Treatment with Ang-II receptor antagonists decreases rates of first and recurrent stroke in hypertensive patients [88], as well as reducing infarct volumes in mice [89]. These findings suggest that a further understanding of B cells in hypertension, particularly antibody production, is needed. The multiple sclerosis (MS) B cell-depleting drug, rituximab, a therapeutic antibody that targets CD20 on the B cell surface to induce apoptosis [90], has already been suggested as a therapy for patients with hypertension but has yet to be tested in the clinic [63, 91].

### Diabetes Mellitus

Type 1 diabetes (T1D) is largely considered to be an incurable autoimmune condition that typically develops during childhood. It is characterized by the destruction of pancreatic insulin-secreting β cells by autoreactive T cells [64, 92]. Diabetes increases the risk of stroke regardless of age [93], and nearly triples the stroke risk in patients with a history of transient ischemic attack [94]. In addition to increasing the risk of stroke, diabetes increases stroke volume and impairs recovery [95, 96].

While T cell-mediated destruction of β cells is undoubtedly important to T1D, B cells are also critical for the development of T1D. Mice that lack B cells or receive anti-IgM therapies do not develop insulitis or diabetes [97, 98], whereas reconstitution of B cells leads to rapid expansion of pathogenic T cells [99]. Multiple methods of pharmacological depletion of B cells delay disease onset, prevent disease development, and induce long-term reversal of disease in mice (see review [90]). In new-onset patients, 4 weeks of treatment with rituximab reduced islet autoantibodies and delayed the decline of C-peptide, a protein produced during endogenous insulin secretion [100, 101]. However, this improvement was transient; by 2 years after therapy cessation, the benefits of rituximab treatment were lost [101]. It has been suggested that greater understanding of the timing and dosing of rituximab during diabetes could improve efficacy [90].

Mechanistically, B cells contribute to diabetes in several ways. MZ and FOB expand during diabetes development [102]. These subsets serve two functions. First, they differentiate into plasma cells to produce autoantibodies against insulin and other pancreatic islet antigens [103, 104]. These autoantibodies trigger a cascade of events, ultimately resulting in increased activation of cytotoxic activity of natural killer cells and CD8 T cells, which, in turn, exacerbates β cell death [103, 105]. In patients with diabetes, the presence of autoantibodies is highly predictive of T1D and often present at high levels at the time of disease onset [104, 106]. Second, MZ B cells migrate to the pancreatic lymph node where they present autoantigens to self-reactive CD4 T cells [102]. Autoreactive T cells from the serum of patients with T1D exhibit enhanced proliferation in response to islet antigens associated with diabetes pathogenesis [107–109]. Interestingly, patient with diabetes also have CNS-reactive T cells that increase the risk of developing CNS autoimmune diseases (e.g. MS [110]).

### Atherosclerosis

Atherosclerosis is a condition in which lipids accumulate in medium to large arteries [111]. Intracranial atherosclerosis occurs when plaques develop in the arteries of the brain and limit blood flow throughout the brain [111]. As of 2004, approximately 70,000 ischemic strokes each year occurred owing to intracranial atherosclerosis, making it a potent risk factor for stroke [112]. Intracranial atherosclerosis is associated with high rates of recurrence, and nearly half of recurrent strokes in these patients are disabling [113]. Atherosclerosis is considered to be a chronic inflammatory disease, with lipid accumulation and adhesion of leukocytes, in particular T cells, linked to arterial inflammation [114]. T cells accumulate in vascular lesions, where they are activated by antigens [115]. In response to activation, they secrete proinflammatory cytokines that worsen inflammation and contribute to disease development [116].

Like many other risk factors for stroke, the role of B cells in atherosclerosis has, until recently, been largely overlooked. The primary B cell “players” in atherosclerosis are B1 and B2 cells, which play protective and pathogenic roles in atherosclerosis, respectively [65]. As mentioned previously, B1 cells are a small population of “innate-like” B cells that produce low-affinity natural antibodies with broad reactivity [23]. While B1 cells are a small population in mice (2–3% in spleen [117]), a similar population of B1 cells (5–10% of B cells in cord and adult blood) has been found in healthy humans [9]. The protective benefits of B1 cells are believed to be predominantly mediated through production of nIgM. In atherosclerosis, nIgM specifically targets epitopes present on damaged lipids, apoptotic cells, and necrotic cells [118]. In patients, high levels of IgM against damaged lipoproteins correlated with slower progression of atherosclerosis and low levels of nIgM against phospholipids increased the risk of stroke [119, 120]. Production of IgM appears to be necessary for protection, as adoptive transfer of IgM-deficient B1 cells into atherosclerotic mice did not reduce lesion size compared with WT B1 cell transfer [121]. In addition to IgM production, B1 cells promote clearance of apoptotic and necrotic debris in lesions [122, 123] and produce IL-10 *in vivo* [25].

In contrast to the protective benefits of B1 cells, B2 cells play a predominantly pathogenic role in atherosclerosis [65]. B2 cells may contribute to pathology, as depletion of B cells using a murine monoclonal anti-CD20 antibody ameliorated disease in 2 different preclinical models of atherosclerosis [124, 125]. It is interesting to note that anti-CD20 treatment selectively depletes B2 cells, while B1 cell populations are left intact [124]. Furthermore, B2-depleted mice have reduced proinflammatory cytokine production [124]. Another study showed that reconstitution of B cells in atherosclerotic mice with B cell-deficient bone marrow worsened disease [126]. While less is known about B2-mediated pathogenesis, there is some evidence that secretion of IgG autoantibodies target epitopes present on damaged lipids, such as plaque antigens, to promote inflammation. These autoantibodies are present in human atherosclerosis lesions [127], and increased levels of IgG are positively associated with disease progression in carotid atherosclerosis [128, 129]. Therefore, a partial explanation for the benefits of B cell depletion may be a reduction in pathogenic IgG autoantibodies [124], but more extensive studies are needed to determine how, if at all, IgM and/or IgG contribute to protection against atherosclerosis and disease pathology, respectively.

## B Cells Also Contribute to Prestroke Neuroprotective Mechanisms

While B cells play a pathogenic role in many stroke risk factors, alterations to B cells also contribute to endogenous protection from stroke injury in animal studies [130, 131]. One method for inducing endogenous neuroprotection against stroke is preconditioning [132, 133]. Preconditioning is a prestroke intervention in which animals or patients receive brief exposure(s) to noxious stimuli that “reprogram” the way the body responds to injury [133, 134]. This reprogramming results in profound protection from stroke in animal models, including reduced infarct volumes, decreased blood–brain barrier disruption, and improved functional and neurocognitive recovery [135–137]. Many preconditioning paradigms have proven efficacious in protecting against stroke, including hypoxic preconditioning, exercise preconditioning, and ischemic preconditioning (see reviews [133, 138, 139]). Interestingly, despite the diversity of preconditioning methods, there is significant overlap in the mechanisms responsible for preconditioning-induced ischemic tolerance [132].

One way in which preconditioning protects is through modulation of the immune system (see reviews [140, 141]). Preconditioning stimuli exert stress on the body, triggering a cascade of inflammatory events that are vital for protection [141–143]. Blocking proinflammatory signaling pathways ablates the benefits of preconditioning on the brain [142–144]. Paradoxically, many of the proinflammatory pathways activated by preconditioning contribute to injury during stroke [141, 145]. Understanding how the immune system contributes to preconditioning-induced neuroprotection may further elucidate the complex interplay between the protective and pathogenic roles of the immune system in stroke. The next section will address how prototypical paradigms of preconditioning (hypoxia, exercise, and ischemia) modulate the adaptive immune system, particularly B cells.

### Hypoxic Preconditioning

Many animal studies demonstrate that a single hypoxic exposure (8–11% O_2_) for 2–4 h induces endogenous neuroprotection (i.e., ischemic tolerance) against transient and permanent strokes [143, 146–151]. Single-exposure hypoxic preconditioning (SHP) protects against stroke by reducing infarct volumes, edema, and neurologic deficits after stroke [151]. SHP significantly modulates the immune system, altering cytokine production, chemokine expression, and overall leukocyte populations [143, 152], including an upregulation of CCL2 in cortical neurons and endothelium [143]. The chemokine CCL2 is responsible for recruitment of leukocytes to sites of inflammation in the CNS [153]. SHP upregulated CCL2 mRNA and protein at 12 h and did not induce neuroprotection in CCL2-deficient mice, revealing a necessary role for this proinflammatory chemokine [143, 154]. However, SHP also altered leukocyte populations in the blood independently of CCL2, including a significant increase in circulating B cells at 12 h after SHP, concomitant with reductions in circulating T cells, monocytes, and granulocytes [143].

**Fig. 3:**
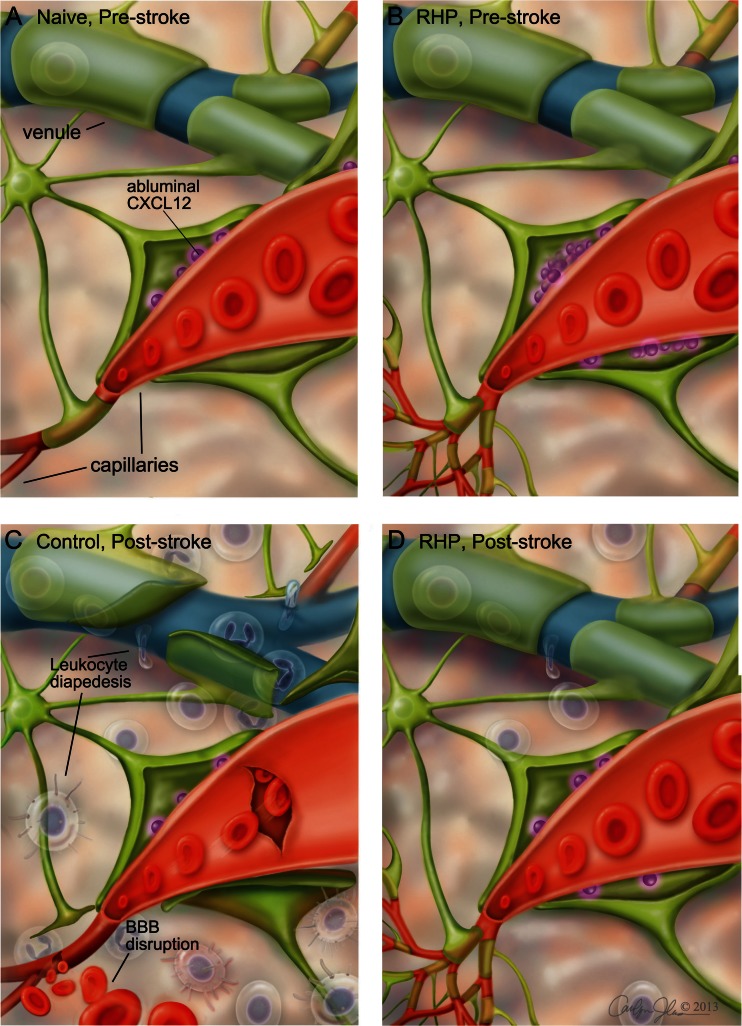
Upregulation of CXCL12 at the blood–brain barrier reduces infiltration of leukocytes

While SHP induces robust protection against stroke, this protection is brief. SHP protects for a maximum of 72 h [146, 150]. However, repetitive hypoxic preconditioning (RHP) greatly extends the window of protection [155]. During RHP, mice receive hypoxic exposures varying in duration (2 or 4 h) and intensity (8% or 11% O_2_) for 2 weeks. RHP reduced infarct volumes by nearly half at 2 weeks after the last exposure, and, remarkably, neuroprotection persisted for 8 weeks following RHP. Despite the global effects of hypoxia, immunomodulation by RHP remained CNS-specific. RHP significantly inhibited diapedesis of CD4 T cells, monocytes, and activated macrophages into the ischemic brain, while recruiting B cells into the injured CNS [130]. Increased B cell representation may be due to an RHP-induced upregulation of vascular CXCL13, a chemokine responsible for B cell recruitment to the CNS during neuroinflammation [130, 156, 157]. A follow-up study found that RHP also upregulates cortical expression of CXCL12, a chemokine that inhibits leukocyte extravasation into the CNS [157, 158]. Blocking post-RHP CXCL12 expression with a CXCL12 receptor (CXCR4) antagonist AMD-3100 disrupted beneficial CXCL12/CCR4 signaling after RHP, and attenuated the anti-inflammatory effect of RHP in the post-stroke brain [130, 158]. While B cells remained elevated in RHP/AMD3100-treated mice, this influx did not exhibit beneficial effects on acute infarct volume. However, higher B cell representation did negatively suppress recruitment of CD4 T cells, which play a pathogenic proinflammatory role in stroke [159]. This confirms the anti-inflammatory effect of RHP-treated B cells, but raises the question of the efficacy on overall neurovascular protection against stroke. Figure 3 depicts the RHP-induced upregulation of CXCL12 at the BBB, and resulting minimizing of BBB disruption, edema, and infiltration of leukocytes in the RHP-treated brain.

While preventative treatment with hypoxia has not been tested in patients with stroke, a longitudinal study from Switzerland found that individuals who were born at higher altitudes, and thus had early exposure to hypoxic conditions, had a decreased risk of stroke [160]. Furthermore, within this population, increasing altitude correlated with decreased mortality from stroke [160]. In healthy individuals, brief repetitive exposures to hypoxia decreased circulating TNF-α for up to 1 week after final exposure, with a concomitant increase in IgG, IgM, and IgA for 4 weeks after the final exposure [161]. Although no clinical studies have specifically examined the effect of repetitive hypoxia in patients with stroke, repetitive hypoxia (alternating 1-min exposure of 9% O_2_ and 21% O_2_ for 15 min) has been used as a therapeutic in individuals with spinal cord injury. This therapy improved somatic motor function, walking speed, and walking endurance [162, 163]. Given impairments in motor function in patients with stroke, it has been suggested that repetitive hypoxia could be used as a therapeutic to improve post-stroke recovery and reduce long-term disability in this patient population [164].

### Exercise Preconditioning

Individuals who exercise regularly exhibit reduced stroke risk, milder stroke injury, and greater functional recovery [165–167]. Exercise is particularly clinically relevant given that it is a noninvasive, easy way to promote protection from cerebral ischemia. Therefore, many clinical studies have investigated exercise as a preventative intervention in individuals at high risk of future strokes (see review [168]). Given the benefits of exercise in clinical populations, animal studies have sought to determine the mechanisms by which prestroke exercise protects against injury and promotes recovery (see reviews [169, 170]). In animal studies, two main methods of exercise are used for preconditioning: forced exercise (short bouts of treadmill running) and voluntary exercise (unrestricted access to running wheels). These two paradigms differentially affect stroke injury and recovery. Forced exercise results in greater reduction of infarct volume, according to a recent meta-analysis, but voluntary exercise in rats leads to greater improvements in motor recovery after stroke [137, 171].

Immunomodulation is a critical component of exercise-induced neuroprotection. Like other forms of preconditioning, ischemic tolerance after exercise preconditioning relies on activation of pro-inflammatory pathways. Over the course of 3 weeks of treadmill running, there is a progressive increase in levels of TNF-α and intracellular adhesion molecule (ICAM)-1, a protein that promotes leukocyte extravasation, in the brains of uninjured rats [172]. However, after stroke, exercised animals have decreased inflammation compared with sedentary controls. Exercise preconditioning decreased levels of endothelial ICAM-1, reduced expression of TNF-α receptors, and diminished leukocyte infiltration into the post-stroke brain [142, 173, 174]. Inhibition of inflammation with a TNF-α antibody in exercised animals reversed improvements in neurological outcome and infarct volume after stroke, supporting previous findings that TNF-α signaling plays a critical role in ischemic tolerance induced by preconditioning [142].

Acute exercise mobilizes B cells through activation of β2-adgenergic receptors by epinephrine, with greater lymphocytosis after high intensity exercise [174]. Similarly, voluntary exercise increases circulating IgM in rats, as well as enhancing B1 cell populations in the peritoneal cavity [175, 176]. In line with this finding, multiple animal studies have found that exercise improves antigen-specific antibody responses and extends the half-life of serum IgG [177–179]. Exercise also reduces apoptosis in splenic B cells isolated from mice that underwent 10 weeks of voluntary wheel running [180]. While alterations in B cell numbers can be difficult to detect in humans, there is evidence that exercise modifies B cell function, specifically increased IgM concentration following mitogen-stimulation (see review [174]). However, the benefits of exercise may be contingent on exercise intensity and duration, as sustained high-intensity exercise suppresses immune function and increases the likelihood of infection in humans [174]. This is of particular relevance as post-stroke infections worsen clinical outcomes and increase the risk of mortality for stroke patients [181].

### Ischemic Preconditioning

Ischemic preconditioning is one of the oldest and most translational paradigms of preconditioning to date. Originally discovered as a way to protect against myocardial infarction in dogs, ischemic preconditioning protects against ischemic injury in other organs, including the brain (see reviews [182, 183]). Ischemic preconditioning can be achieved by 1 of 2 methods: cerebral ischemia or remote limb ischemia. Cerebral ischemic preconditioning is a method used in rodent studies in which the animal is given either a brief focal ischemic exposure or global ischemic exposure [141]. A transient occlusion of the femoral, mesenteric, or renal artery in animals, for example, induces remote ischemic preconditioning (rIPC) [169]. In clinical populations, the typical protocol for rIPC is 3 to 5 inflations of a blood pressure cuff to 200 mmHg on the forearm or leg, separated by brief periods of reperfusion [184–190]. rIPC has since been used as a safe, noninvasive method to reduce injury during coronary artery bypass surgery, abdominal aortic injury repair, pediatric cardiac surgery, and liver resection, as well as to enhance athletic performance and motor learning in healthy individuals [191–195]. Given its efficacy in decreasing ischemia–reperfusion injury, there is significant interest in application as a pre- and post-stroke intervention during ischemic stroke [196], especially in light of promising results using rIPC in other CNS diseases [187–190]. However, some clinicians express concern about our incomplete understanding of the mechanisms behind ischemic preconditioning-induced neuroprotection [190].

Our knowledge of the mechanisms of neuroprotective immunomodulation by cerebral ischemic preconditioning is limited, hindering our understanding of how B cells contribute to immune-mediated neuroprotection [141]. Ischemic preconditioning upregulates IL-1β protein, a proinflammatory cytokine that can induce B cell activation and antibody production [197, 198], and activates Toll-like receptor (TLR)-4 increases B cell proliferation, migration, and immunoglobulin secretion [199]. rIPC increases B cell populations after stroke in rats [131], mirroring our findings that B cell populations are enhanced following RHP in healthy mice. Interestingly, these findings contradict studies surrounding preconditioning that largely describe activation of acute proinflammatory signaling pathways [141], as both rIPC and RHP have, instead, an anti-inflammatory effect on the immune system that may unique to repetitive preconditioning stimuli [134].

## The Role of B Cells in Stroke Injury and Recovery

Immediately after stroke there is tissue damage due to absence of blood supply to the cells. Following primary cell death, BBB integrity is compromised, with secondary reperfusion injury due to the infiltration of immune cells and the ensuing inflammatory cascade [200]. Activated endothelial cells lining the BBB express adhesion molecules to recruit peripherally circulating immune cells to bind to the endothelium and infiltrate into the brain parenchyma. This immediate and early pro-inflammatory response to stroke is mainly mediated by innate immune cells (i.e., not antigen specific) and induces cell death, tissue damage, and behavioral dysfunction [201]. This section will summarize what is known about B cell-mediated mechanisms in post-stroke injury and recovery.

### CNS Immune Responses after Stroke

Infiltrating immune cells and activated tissue resident immune cells (e.g., microglia) constitute immediate, acute post-stroke inflammation [202]. In this acute phase, lymphocytes like B cells and T cells secrete cytokines and produce reactive oxygen species without regard to antigen specificity [203, 204]. Molecular signals known as danger-associated molecular pattern molecules, released by the damaged and dead neurons and other cells in the ischemic hemisphere, bind to TLRs, scavenger receptors, and other receptors on the surface of APCs for activation [205]. Dead and dying cells break down within the ischemic area within hours after stroke onset [206, 207]. Owing to BBB disruption, these cell fragments, which are normally sequestered from the peripheral circulation, act as CNS-specific antigens and elicit delayed, antigen-specific adaptive immunity after stroke [208–211]. Primed APCs present these CNS antigens to T cells and B cells in both the draining cervical lymph nodes and the spleen, and initiate the development of an adaptive immune response specific for the CNS antigens [212]. Even though by central and peripheral tolerance mechanisms most autoreactive cells have been eliminated, a few still escape into the periphery and respond to the CNS antigens [208–210, 213]. However, these post-stroke antigen-specific immune responses are largely undefined. Additional studies are required to understand if they potentiate further CNS tissue damage following stroke [208, 214–216], particularly owing to the recent identification of lymphatic vessels within the meninges [217, 218].

### Post-stroke Protective Effects of B Cells

There is no clear consensus on the role of B cells in post-stroke recovery, though several animal studies propose a protective role for B cells in neuronal injury following stroke. μMT^–/–^ mice, which have a nonsense mutation introduced into the transmembrane exon of the IgM heavy chain resulting in the total deletion of B cells [219], have larger infarct volumes, worse functional deficits, and a higher mortality rate after middle cerebral artery occlusion [220]. The ischemic hemisphere also exhibited higher numbers of activated T cells, macrophages, microglial cells, and neutrophils than WT mice. Prestroke adoptive transfer of highly enriched populations of WT B cells to μMT recipient mice was protective. Protection may be contingent on cytokine secretion, as the same neuroprotective phenotype was not observed when IL-10-deficient B cells were adoptively transferred prior to stroke [220].

Mice receiving B cells as a neurotherapeutic exhibited higher numbers of peripheral regulatory cells concomitant with lower numbers of activated pro-inflammatory T cells [221]. Regulatory T and B cells attenuate post-stroke inflammation by producing anti-inflammatory cytokines (e.g., IL-10, TGF-β). During CNS pathology, IL-10 expression in the brain increases regulatory lymphocytes and reduces CNS inflammation [51, 222–224]. LPS stimulation of B cells prior to adoptive transfer enhances IL-10 production and reduces infarct volumes, increases regulatory T cell numbers, and attenuates peripheral pro-inflammatory responses [224]. Given glatiramer acetate (i.e., copaxone) increases Breg populations in the blood and production of IL-10 [225], it has been investigated in preclinical studies of focal stroke. While Ibarra et al. found that glatiramer acetate reduced post-middle cerebral artery occlusion infarct volumes [226], this neuroprotective result has been questioned by lack of efficacy in other murine studies [227].

### Post-stroke Detrimental Effects of B Cells

Other animal studies have indicated B cells as either uninvolved in, or actively injurious during post-stroke functional recovery. Mice deficient in lymphocytes [i.e., recombination activating gene (Rag1)^–/–^], or only deficient in CD4 T cells, CD8 T cells, B cells, or IFN-γ, were used to determine the contributions of specific lymphocyte populations to ischemia–reperfusion injury and recovery. The Rag1^–/–^ mice had lower infarct volumes and reduced neurological deficits, but B cell-deficient mice failed to show improvements post-stroke, suggesting B cells did not significantly contribute to infarct progression [228]. Furthermore, reconstitution of RAG1^−/−^ mice with B cells did not significantly induce post-stroke neuroprotection [229].

The occurrence of stroke increases susceptibility to dementia, in particular vascular dementia, with respect to other risk factors like diabetes, hypertension, age, and hypercholesterolemia [230–234]. However, the signaling pathways leading to post-stroke dementia development are not completely understood. Recent data report the development of cognitive deficits 7 weeks after stroke in mice [233]. In correlation with cognitive deficits, B cells aggregated in the infarct region 4 to 7 weeks post-stroke, and produced IgA and IgG antibodies. This has been verified in 4 stroke models in 2 different mouse strains. Patients with stroke also demonstrate immunoglobulin synthesis in the cerebrospinal fluid for months after stroke [235–237]. These antibodies may result in neuronal damage and further cognitive impairment by binding to the Fc receptors and activating the complement pathway, as seen in lesions of patients with MS [238]. Moreover, these antibodies could exacerbate disease by egressing into the adjacent, unaffected healthy tissue.

B cell antibody production can also directly alter neuronal function. Antibody accumulation correlated with impairments in hippocampal long-term potentiation, resulting in short-term memory deficit weeks after stroke [233]. Additionally, μMT mice did not develop delayed cognitive deficits, and rituximab attenuated cognitive decline. As opposed to the beneficial role of B cells for neuroprotection in the acute phase of stroke, these lymphocytes may play a detrimental role in post-stroke long-term cognitive impairment [233, 239]. Further studies will elucidate the dependence of CNS injury on the magnitude, location and timing of B cell subsets, in order to determine whether B cells induce neuroprotection, supporting functional plasticity, or exacerbate post-stroke recovery.

## Current Stroke Clinical Trials That Potentially Impact B Cell Function

Of the clinical trials related to acute ischemic stroke, only a select few exhibit potential B cell implications in stroke. Table 1 summarizes the existing stroke clinical trials that provide reasonable sources of speculation for the role of B cells in functional recovery. To begin, several FDA-approved therapies for the treatment of MS, including fingolimod, natalizumab, and IFN-β, are now being applied to patients with acute ischemic stroke (AIS) [240]. A phase II clinical trial of natalizumab (Table 1, study 1) that assess efficacy in patients with AIS was recently completed, though the final data have not been released. However, evidence indicates that natalizumab can have a suppressive effect on B cells by inhibiting functional antibody synthesis in patients with MS [241], and thus the effect of natalizumab on B cells during AIS should be investigated. Fingolimod is now in a recruiting phase II clinical trial (Table 1, study 2) as a treatment during AIS [242]. In MS and intercerebral hemorrhage studies, fingolimod improves neurological function [243], reduces memory B cells [244], and promotes Breg phenotype and function [245]. A completed phase I clinical trial using IFN-β, with results unavailable (Table 1, study 3), investigated its safety in AIS. IFN-β knockout mice exhibit increased B cell proliferation in the blood, weight loss, and reduced strength post-stroke [246], indicating that IFN-β may attenuate inflammation in a manner related to B cell activity.

**Table 1:** Clinical stroke trials related to B cells

Clinical study	Trial ID/status	Purpose/aim	Methodologies
Effect of Natalizumab on Infarct Volume in Acute Ischemic Stroke (ACTION)	NCT01955707/phase II: completed	Assess natalizumab efficacy on change in: • infarct volume • clinical measures of stroke outcome • safety in patients with AIS	• Brain MRI • mRS
Efficacy and Safety of FTY720 for Acute Stroke	NCT02002390/phase II: currently recruiting	Test immune modulation of fingolimod in AIS	• Brain MRI • Flow cytometry of IV blood
Safety Study of Interferon Beta 1a to for Acute Stroke	NCT00097318/phase I: completed	Investigate the safety of IFN-β1a in patients with AIS	• Brain MRI/CT • Blood tests (serum)
The IMPULSE Study: Pilot (IMPULSE)	NCT02044471/currently recruiting	Investigate if ischemic conditioning will lower blood pressure	• Monitor blood pressure • Monitor inflammatory markers: TNF-α, IL-6, IL-8, IL-10
Protective Effects of Remote Limb Ischemic Preconditioning on Acute Cerebral Infarction	NCT01672515/phase I/II: not yet recruiting	Investigate whether postconditioning can reduce infarct volume of patients with ischemic stroke	• Remote ischemic postconditioning • Assess serum CRP, TNF-α, ICAM-1, and GFAP
Immunological Biomarkers in Patients With Acute Ischemic Stroke	NCT01894529/completed	Link immune markers previously associated with AIS clinical outcomes as potential biomarkers to tailor therapy	• Serum cortisol and IL-10 • Circulating B cells • Monocyte TLR-4, HLA-DR, CD86, and VLA-4 expression • *Ex vivo* TNF-α production
The Role of HMGB-1 in Chronic Stroke	NCT01705353/currently recruiting	Measure blood HMGB-1 at different time points after stroke to determine if its presence correlates with rate of stroke recovery	Measure serum cytokine levels
White Blood Cell Counts and Onset of Cardiovascular Diseases: a CALIBER Study (CALIBER)	NCT02014610/active: not recruiting	Investigate if particular types of WBC types are associated with a range of cardiovascular diseases (including stroke)	Measure lymphocytes, neutrophils, eosinophils, monocytes, and basophils
Inflammation and Post-stroke Depression	NCT02368145/ongoing: not recruiting	Determine if a relationship exists between stress/inflammatory blood compounds and the presence, absence, or degree of depression in patients with AIS	• Measure blood proinflammatory cytokines/glucocorticoids • Measure depression

AIS = acute ischemic stroke; MRI = magnetic resonance imaging; mRS = modified Rankin Score; IV = intravenous; IFN = interferon; CT = computed tomography; TNF = tumor necrosis factor; IL = interleukin; CRP = C-reactive protein; ICAM-1 = intercellular adhesion molecule 1; GFAP = glial fibrillary acidic protein; TLR = Toll-like receptor; HLA-DR = human leukocyte antigen–antigen D-related; VLA-4 = very late antigen 4; HMGB-1 = high mobility group box 1; WBC = white blood cell

The third part of this review highlights studies that use preconditioning to minimize post-stroke injury, potentially through modulation of the adaptive immune system. Currently, several clinical trials are investigating if ischemic conditioning can decrease blood pressure (Table 1, study 4), or if postconditioning reduces infarct volume in patients with AIS (Table 1, study 5). Both studies aim to monitor serum levels of inflammatory markers that are potentially relevant to B cells, such as TNF-α, IL-6, IL-8, and IL-10, or TNF-α, ICAM-1, and glial fibrillary acidic protein, respectively. Interestingly, the interaction of LFA-1 and ICAM-1 on B cells lowers B cell activation levels [247, 248].

“Immunological Biomarkers in Patients With Acute Ischemic Stroke” is a completed clinical trial that focused on discovering immunological biomarkers in AIS in order to tailor therapy to ameliorate negative stroke outcome (Table 1, study 6). They measured circulating B cells, serum cortisol, IL-10 levels, monocyte expression of TLR4, human leukocyte antigen–antigen D-related (HLA-DR), CD86, a co-stimulatory molecule necessary for T cell activation, and very late antigen 4, an integrin that regulates the migration of leukocytes to sites of injury. When the results are published, this study will shed light on how variations in circulating B cells and their secreted cytokines can serve as biomarkers of functional recovery [249]. A clinical trial is investigating the role of serum cytokine, high motility group box 1 (Table 1, study 7). High motility group box 1 is primarily produced by mature dendritic cells to activate B cells and increase T cell reactivity, including the promotion of autoreactive B cell responses to TLR-9 ligands and DNA immune complexes by T cells [250, 251].

Associating specific white blood cell types to the pathology of AIS could ultimately allow for optimal targeted therapy, and is therefore being pursued in the CALIBER trial (Table 1, study 8). White blood cell counts can predict disease progression and outcomes of AIS [252, 253], with evidence of reduced lymphocyte circulation (B cells included) in the blood of patients following stroke [254]. Unfortunately, no clinical studies show a link between AIS, cognitive decline, and B cells, though an ongoing clinical trial (Table 1, study 9) seeks to uncover the possible relationship between inflammatory/stress compounds and post-stroke depression. It is possible that CNS-derived B cells release cytokines or antibodies that are relevant to depression [255], which will hopefully be elucidated in future clinical trials.

## FDA-Approved B Cell Targeting Therapies Used in Other CNS Diseases

As described in this review, there is clear evidence in both human and mouse studies that B cells can either mediate or suppress stroke-induced pathology. By understanding the role of B cells in other CNS diseases, we may discover how B cells may truly benefit the recovering brain after stroke. In the clinic, therapies targeting B cells are used to treat CNS diseases, particularly the prototypical CNS autoimmune disease, MS. Rituximab was approved by the FDA in 1997 for follicular non-Hodgkin’s lymphoma. More recently, rituximab was found to be efficacious in patients with relapsing–remitting MS [18, 256, 257], and also reduces neurological dysfunction in the primary progressive form of MS [258].

B cell depletion successfully treats another CNS disease, anti-*N*-methyl-D-aspartate receptor (NMDAR) encephalitis, which is caused by antibodies directed against the NR1 subunit of the NMDAR [259]. Patients with anti-NMDAR encephalitis may have neurological disturbances such as seizures, catatonia and coma [260, 261]. First-line treatment includes the use of cortisteroids, plasmapheresis (i.e., promotes removal of circulating autoantibodies), and intravenous administration of immunoglobulin in order to elicit an immuno-modulatory effect. A combination of rituximab and cyclophosphamide may be used as secondary treatment to deplete all circulating B cells and induces a generalized immune-suppressive effect, but initiation of clinical trials has not yet begun [261, 262]. Through studying B cell therapies for other CNS diseases, we may be able to differentiate between pathogenic and therapeutic B cells and determine how to promote the latter, resulting in effective CNS therapies in stroke.

## Conclusions

The “common soil” hypothesis, first mentioned in the second section, suggests that shared disease mechanisms for hypertension, diabetes, and atherosclerosis as the greatest risk factors for stroke [75, 76]. B cell functions, in particular antibody production, are the perfect candidates to mediate these shared mechanisms. However, as outlined in this review, this commonality could also extend to protective B cell mechanisms that create an endogenous ischemic tolerance to ultimately reduce stroke injury and promote repair. Understanding how the adaptive immune system shifts from harm to benefit, and particularly which B cell functions in the at-risk or injured CNS should be therapeutically attenuated, could prove efficacious in treating a disease that affects millions globally on an annual basis.

## Electronic supplementary material

Below is the link to the electronic supplementary material.

ESM 1 (PDF 1224 kb)
